# Data availability on men's involvement in families in sub-Saharan Africa to inform family-centred programmes for children affected by HIV and AIDS

**DOI:** 10.1186/1758-2652-13-S2-S5

**Published:** 2010-06-23

**Authors:** Victoria Hosegood, Sangeetha Madhavan

**Affiliations:** 1 Centre for Population Studies, London School of Hygiene and Tropical Medicine, UK; 2 Africa Centre for Health and Population Studies, University of KwaZulu-Natal, South Africa; 3 African American Studies Department, University of Maryland, USA; 4 Medical Research Council/Wits Rural Public Health and Health Transitions Research Unit, School of Public Health, University of the Witwatersrand, South Africa

## Abstract

The Joint Learning Initiative on Children and AIDS recently recommended that programmes for children affected by HIV and AIDS in sub-Saharan Africa direct more support to families. Interest has grown in including men in such family-orientated interventions by researchers, policy makers, and community and non-governmental organizations. However, there is a lack of good quality data on men's involvement with children in the diverse settings in sub-Saharan Africa. In addition, limited research has examined their role in providing emotional, material support and protection for HIV- and AIDS-affected children and families.

In this paper, we describe the availability of data about men and families, in particular fathers, in ongoing sub-Saharan African surveys and longitudinal population cohorts. We discuss the conceptual and measurement issues associated with data collection on men's involvement in these types of studies. We consider the opportunities for improving the collection of data about men and families in household surveys and population cohorts in order to inform the design and evaluation of family-centred interventions for children affected by HIV and AIDS.

## Introduction

The Joint Learning Initiative on Children and AIDS has recently recommended that families need to be more central in intervention programmes to support children affected by HIV and AIDS in sub-Saharan Africa [1]. The growing body of evidence about the unique contributions that biological and social fathers make to child health, welfare and other outcomes [2-4] has encouraged researchers [1,5-12], policy makers [13], and community and non-governmental organizations [14,15] to explore how men might be engaged in family-centred interventions.

Efforts to develop effective interventions that promote positive involvement by men in the care and support of children face a number of challenges. Although considerable experience has been built up about how to promote maternal involvement with children through interventions, far less is known about how to support the positive involvement by fathers and other men within families. Given the wide variation in family forms and family functioning that exist in sub-Saharan Africa, there are good reasons to anticipate that the levels and types of men's involvement will vary considerably, as will the ways in which this can be promoted and supported [16-18]. Within the region, differences also exist in severity of the HIV epidemic, the impacts on families and households, and the wider economic and political contexts [19].

The importance for family policy and programmes of comprehensive, appropriately conceptualized, reliable and valid data on men's involvement is illustrated by the experience of the United States. Detailed data on fathers has been collected by the National Study on Family Growth [20], the National Longitudinal Study of Youth [21], and the Fragile Families project [22,23]. These data have been used to inform the development of effective policies to support low-income (particularly African-American) fathers and their families [24]. In sub-Saharan Africa, the high cost of specialized family studies means there is a greater reliance on alternative sources of data about men and families [25].

In this paper, we consider the availability of empirical data about men's involvement with families in ongoing surveys and longitudinal population cohorts in sub-Saharan Africa. We focus particularly on the identification of men who are fathers and information about how they contribute to, and are involved with, their children. We highlight conceptual and methodological issues related to data collection, and suggest ways in which data might be improved to inform the design and evaluation of family-centred interventions that engage men in the support of children affected by HIV and AIDS.

We begin our paper by briefly reviewing the socio-demographic impact of HIV and AIDS on families and households and the evidence for men's involvement in affected families. We describe the types of data available about men and families, and discuss the conceptual and measurement issues associated with data collection. We highlight areas for strengthening the availability of data that can be used to inform and evaluate family-centred interventions for children affected by HIV and AIDS. Our paper complements another paper in this issue by Lorraine Sher, which discusses fathering in the context of HIV and AIDS.

## Discussion

### HIV impact on children and families

Families and the households in which they live are central in shaping the health, development and wellbeing of children [26,27]. In sub-Saharan Africa, the HIV epidemic has placed a severe burden on families over the past two decades, many of which have also faced a wide range of other concomitant social, economic and political adversities.

Recent review papers have examined the evidence for the socio-demographic impact of the HIV epidemic on families and households within the region with respect to living arrangements, fertility, mortality, migration, union formation, and household developmental lifecycles [28,29]. The cumulative impact of HIV and AIDS on families extends beyond the direct demographic and economic impacts of illness and death to encompass the effects on psychosocial wellbeing of indirect consequences of the HIV epidemic, including stigma, grief and family dispersal (for overviews see [1,19]).

The prevalence of all types of orphaning has risen substantially in sub-Saharan Africa, mirroring the increases in adult mortality since the start of the epidemic [30-32]. Empirical multi-country comparative studies using cross-sectional data have most consistently found orphans to be at risk of poorer education outcomes than non-orphans [33], whereas for other outcomes, including growth and malnutrition, the findings are more mixed [34].

The ability of many families to care for and support children has undoubtedly been threatened by the HIV epidemic. The published data and empirical literature, however, consistently show that most affected families and households in sub-Saharan Africa adapt and continue to provide for the needs of children [1]. The majority of orphans have a surviving parent, and most children affected by HIV and AIDS live with parents and other adult family members [30,31]. Multiple studies have shown that the proportion of child-headed house-holds remains small, even in severely affected communities [31,35,36]. One aspect that has received less attention is the role of men in family responses to HIV/AIDS.

### Men and families affected by HIV and AIDS in sub-Saharan Africa

Family formation remains strongly linked to childbearing and marriage, even though the domestic arrangements that result are heterogeneous and dynamic in different cultural, demographic, economic and political contexts [37]. These contextual factors influence the way in which family life is organized with respect to membership, roles and responsibilities, including parenting and child care [18,38]. Furthermore, although cultural and social norms specify normative behaviours of men in relation to their own biological children and other children in the family, specific circumstances (e.g., labour migration, extra-marital fertility, multi-partner fertility, divorce or maternal deaths) may lead men to establish new social and residential arrangements regarding children or take on new or modified roles and responsibilities.

Studies investigating family responses to the care and support of children affected by HIV and AIDS in sub-Saharan Africa have focused almost exclusively on the role of mothers, grandmothers and other female relatives. Where data are collected about fathers, the emphasis is typically on financial contributions. Few studies collect information about the family roles and responsibilities of men, other than those of biological fathers.

Despite, or perhaps because of, the limited amount of detailed data about men's involvement in other activities related to children, the findings bolster assumptions about the absence or limited involvement of men, particularly fathers. Assumptions about the involvement of non-resident fathers and other men have been challenged by findings from qualitative studies of men and families in southern Africa [18,39-42].

Re-examining data from an ethnographic study of households in rural South Africa that had experienced adult AIDS illness or deaths, Montgomery *et al *(2006) found that men were positively involved with their families and households in a wide range of ways, including caring for people who were ill, caring for children, undertaking domestic activities, and financially supporting immediate and extended family members. However, the involvement of men in these activities was not readily acknowledged by female respondents or men themselves, nor anticipated by interviewers.

Studies have also shown that men's involvement needs to be understood as part of a kinship network that seeks to meet the needs of children [16,38]. These networks include men; therefore, when biological fathers are unable to meet the needs of their children, their own fathers or brothers may step in and assist. In the context of HIV and AIDS, support to HIV-infected fathers who become ill includes assistance in fulfilling paternal roles and responsibilities [43].

The HIV epidemic has been most severe in southern Africa, where several distinctive family and household characteristics (albeit not unique or universal) have implications for the design of effective family-based interventions that engage fathers and other men. A combination of historical and contemporary social, historical, political and economic factors have resulted in high levels of residential separation of biological fathers and their children. Some of these factors, for example, the apartheid political system and its effects on labour migration, settlement and family separation, are specific to South Africa and neighbouring countries. Others, such as urbanization and increasing marital instability, are increasingly influencing men's experience of family life in other parts of the region.

In southern Africa, many households function as "stretched" residential units with family members "dispersed" between different households [44,45]. Low rates of marriage [46,47], together with cultural norms related to household formation and childbearing, also contribute to the social and residential separation of biological fathers from their children [41]. The majority of young children born to unmarried parents will live with their mothers [48,49].

### Data about fathers and children available from survey and population cohorts

The most widely available sources of demographic data on families and households in the region are the Demographic and Health Surveys (DHS) conducted in most countries in the region [50]. Sources of detailed data on sub-national populations are the ongoing Demographic Surveillance Systems (DSS) conducted in several African countries [51]. In addition, there are several ongoing child cohorts and household panel studies that collect data on family structure, parenting, and experiences of HIV and AIDS [52-54]. Currently, surveys and population cohorts collect very limited data about men's involvement with children and families [55]. In this section, we describe commonalities in the available data on men and fathers. We also consider several conceptual and methodological issues related to the types of data needed to inform the design and evaluation of family-based interventions for children affected by HIV and AIDS.

### Identity and characteristics of biological fathers

Information about the identity and survival status of children's biological fathers is collected by most household surveys and population cohorts. The identification specifically of biological fathers is not always clearly specified. While the identity of children's fathers is typically restricted to men who are listed in the household roster, most surveys collect information about paternal orphaning for all children in the household. This is usually done by simply asking a household respondent whether the father of each child is alive. Data on paternal orphanhood is used in research studies as a potential risk factor for health and welfare outcomes in children, but can also be used to estimate adult mortality [56].

Where fathers are co-resident household members, information commonly available includes his age, education, employment and marital status. The co-residential arrangements of children and their fathers are documented by surveys and cohorts. However, information about living fathers who are not members of the same household as their child is not usually available.

In household surveys and demographic surveillance systems, the primary sampling and enumeration unit is the household, rather than families. Therefore, specific questions must be asked to establish the identity, characteristics and involvement of fathers living in other households, questions that few large sub-Saharan African surveys or longitudinal studies ask at present. Engaging fathers is a challenge for all family interventions, particularly when fathers are not co-resident with their children, and it is important to understand the specific circumstances in which fathers live apart from their children.

### Social fathers

It is important for family research and interventions that information about men's involvement with children is not restricted solely to biological fathers. The person fulfilling the role of father may not always be the child's biological father. Social fathers, a term that includes stepfathers and foster and adoptive fathers, are a common feature in sub-Saharan Africa social and cultural contexts [16,57]. In matrilineal societies, social fathering will often be the responsibility of a child's maternal uncle, even when his or her biological father is living. Men may take on a social fathering role for the children of new partners, for younger siblings or for grandchildren.

Fathering roles may also be performed by women, for example, in situations where children are raised by single mothers or grandmothers. The phenomenon of social fathering is exacerbated by high rates of labour migration, union instability and orphaning due to paternal AIDS deaths. Unfortunately, despite the strong justification that collecting data about social fathers provides a more complete picture of fathering and social protection, such information is seldom collected in sub-Saharan surveys or population cohorts.

### Men's involvement in families of children affected by HIV and AIDS

The measurement of involvement by biological and social fathers has been the subject of considerable multi-disciplinary attention [3,4]. Central components of father involvement include paternal engagement, accessibility and responsibility, including economic contributions [58]. However, with the exception of specialized family studies, very limited data about men's involvement is collected by surveys and population cohorts.

Commentators have suggested that the lack of data collection is a reflection of the normative attitudes and stereotypes on the part of researchers and policy makers, who consider African fathers to have limited engagement with children [17]. Information about men's involvement is almost exclusively restricted to questions about biological fathers' co-residence and financial support. However, in South Africa, qualitative research has shown that co-residence of fathers with children is a poor indicator of men's involvement with children [59,60].

In surveys and cohorts, data about father involvement can potentially be collected from the perspective of the child or the father. Each adult man in the household can be asked about his involvement with each child in the household or with any child outside the household. For each child in the household, the type of involvement that his or her biological or social father has can be specifically documented. However, these approaches are rarely used in surveys.

Rather, the more commonly used method is to ask a household respondent to identify which person has "main" or "primary" responsibility for a child with respect to a specific activity, for example, care giving or payment of school fees. Should this person not be the child's father, any involvement by the father in these activities will be unrecorded. One exception is the National Income Dynamics Survey, a South African panel survey that has collected information about financial contributions by biological and social fathers to children within and outside study households [61].

Survey data about men's involvement with children and families in surveys could contribute greatly to the design and evaluation of interventions that seek to engage men in family- or school-based interventions. In longitudinal cohorts, information about father involvement can also be used as a screening tool to: identify children who lack positive support and protection by men within their families, for example, paternal orphans living without other male kin; or to identify positively involved men who may benefit from additional support, for example, co-residential fathers following the death of the child's mother. Routinely collecting data about father involvement with children affected by HIV and AIDS in longitudinal population cohorts may also provide a cost-effective approach to monitoring and evaluating family-based programmes.

### How can data collection be improved?

The experience of fatherhood scholarship in developed countries has been that social surveys can be used to collect information about the kinds of activities that resident and non-resident fathers, as well as other men, engage in with respect to children of different ages [62]. However, enhancing the collection of family data in ongoing studies in sub-Saharan Africa requires a balance between the benefits of the additional family data and constraints due to the design and cost of large surveys and population cohorts. One of the benefits of population-based data is the ability to document the way families exist and function in the real world as opposed to the more controlled environment of intervention studies.

However, data collection in nationally representative household surveys and large population cohorts typically rely on proxy reporting. This has implications for data reliability and validity as proxy reporting may lead to selective bias in reports of men's involvement with children and families. For example, family respondents tend to under-report financial contributions by non-resident fathers [63,64].

Undoubtedly, a central challenge to improving data collection on men's involvement in sub-Saharan Africa, and most especially in southern Africa, is the extent of residential separation of men, children and families. The social and economic rationales for including resident and non-resident household members in household surveys has been recognized in the design of many surveys in countries with high levels of migration, for example, South Africa [49,65,66].

It is reasonably straightforward to ask whether each man is involved in activities related to each child in the household. Basic characteristics of these men are already collected as part of the survey. However, information about any contributions or involvement by men that are not listed on the household roster will have more value for research if it is linked with other data about the man, for example, the type of relationship he has with the child and the child's mother, and his socio-demographic characteristics. This data would usually need to be obtained from a proxy respondent.

Interviewing men themselves may also be a strategy in enhancing data on men's involvement. This option is particularly attractive in household surveys that already administer adult questionnaires, for example, to collect data on income or reproductive health. Sampling of men from the household roster would not include fathers or other involved men outside the household. Family studies have shown that it is possible to contact and interview "hard-to-reach" non-resident fathers, although this can be very resource intensive and subject to gatekeeping by household members, particularly mothers [67]. Were surveys to collect data on father involvement from proxy respondents and men themselves, it would be important to examine the reliability and validity of multiple sources of data [64].

In summary, data collection to support intervention research can be improved by: (i) collecting information about the identity and involvement of social and biological fathers within and outside the study household; (ii) extending data collection efforts to include non-resident fathers and other family members given the context of dispersed families and high levels of migration in sub-Saharan Africa; (iii) collecting information that reflects the inter-dependence of family members and the existence of multiple family environments providing care and support to children; (iv) assessing the reliability and validity of data about fathers and father involvement reported by proxy household respondents; and (v) collecting paternity histories.

### Data to inform family-centred programmes for children affected by HIV and AIDS

Survey data about men's involvement with children and families in surveys could contribute greatly to the design and evaluation of interventions that seek to engage men in family- or school-based interventions. For the design and evaluation of family-centred programmes to support children affected by HIV and AIDS, there are several key indicators related to men's involvement with children and families that could feasibly be collected by many of the ongoing surveys and population cohorts in sub-Saharan Africa. These include:

For each child:

◆ Identity of biological father

◆ Identity of social father (e.g., stepfather, foster father, grandmother)

◆ Identity of mother's partner (if not in a fathering relationship with the child)

◆ HIV and AIDS experiences:

◆ HIV infection (self, parent, other household member)

◆ AIDS illness and mortality (parent, other household member)

◆ Child health, development and wellbeing indicators

◆ Biological and/or social fathers' involvement:

◆ Co-residence with his child

◆ Time spent with child, frequency of visits

◆ Father's activities by type and time with child (care, meals, play)

◆ Quality of relationship between father and child

◆ Quality of relationship with child's biological mother

◆ Quality of relationship with child's primary caregiver (if not mother or self)

◆ Financial or material support for child by type and amount

◆ Financial or material support for household by type and amount

◆ Involvement by other resident or non-resident men (not father of the child):

◆ Financial or material support to child and household

◆ Relationship of child to other men who contribute or are involved with child

For each biological or social father:

◆ Survival status of father (date of death, age at death)

◆ Place of residence

◆ Demographic characteristics (age, residential patterns, marital and partnership status, ethnicity, language, education)

◆ Social characteristics (relationship to other household members)

◆ Economic characteristics (employment status, income)

◆ Health (general health status, mental health, alcohol and drug use)

◆ Paternity history with identification of child's mother and survival status of mother

## Conclusions

Family-based interventions can be used to support HIV- and AIDS-affected children and families in a range of different ways. These include: family-based HIV testing delivered at home; HIV prevention programmes that involve parents and children; family treatment support for HIV-infected children and adults, including case management and service delivery; and programmes to support families of HIV- and AIDS-affected children with financial assistance (for education, housing, food), counselling and medical care.

Successful interventions will be those that build on the strengths of family functioning by developing models based on a knowledge about how families provide care and support to children, and develop appropriate models of delivery suitable in varied social, economic and infrastructure contexts [68]. For example, interventions that recognise inter-household, as well as intra-household relationships and involvement, will be better able to support those children affected by HIV and AIDS whose families are dispersed and where the men that support them are not co-resident.

The development of culturally appropriate, safe and acceptable family-centred interventions that can successfully engage men in the support of HIV- and AIDS-affected households requires detailed family data. For example, programme delivery should consider men's presence patterns, recognising that non-resident fathers may not be able to participate in home- or school-based programmes. Data about men's paternity and partnership histories will assist in understanding barriers to increasing men's involvement.

The enrolment of fathers or other male relatives may sometimes be impossible or ill-advised, as in the case where these men are in prison or hospital, or have problems related to mental health, drugs or alcohol, or have physically or sexually assaulted members of their family [69]. This issue may be particularly pertinent in such countries as South Africa, where high rates of domestic violence and child sexual abuse have been reported [70].

Ongoing surveys and population cohort studies in sub-Saharan Africa are not only valuable sources of data on men and families, but could be used as tools for evaluating family-centred interventions. A recent systematic review by King *et al *(2009) identified no rigorously evaluated studies of health and welfare family interventions to improve the psychosocial wellbeing of children affected by HIV and AIDS in sub-Saharan Africa [71]. The urgent need for evaluation studies is another impetus to improve the availability of data about men and families, particularly in population and community cohorts whose longitudinal design makes them ideally suited as platforms for family intervention research.

## Competing interests

The authors declare that they have no competing interests.

## Authors' contributions

VH and SM drafted the manuscript and approved the final version.

## References

[B1] Richter LM Sherr L Adato M Belsey MA Chandan U Desmond C Drimie S Haour-Knipe M Hosegood V Kimou J Sangeetha M Mathambo V Wakhweya A Strengthening families to support children affected by HIV and AIDS AIDS Care 2009 13 3 12 10.1080/09540120902923121 22380973 PMC2903779

[B2] Lamb ME The Role of the Father in Child Development 2004 Fourth New York: Wiley

[B3] Lamb ME, Day RD Conceptualizing and Measuring Father Involvement 2004 Mahwah, NJ: Lawrence Erlbaum Associates

[B4] Tamis-LeMonda CS, Cabrera N Handbook of Father Involvement. Multidisciplinary Perspectives 2002 Mahweh, New Jersey: Lawrence Erlbaum Associates, Inc

[B5] Green CP Cohen SI Belhadj-el Ghouayel H Male Involvement in Reproductive Health, Including Family Planning and Sexual Health 1995 New York: UNFPA

[B6] Helzner J Men's involvement in family planning Reproductive Health Matters 1996 13 146 154

[B7] Mbivzo MT Bassett MT Reproductive health and AIDS prevention in sub-Saharan Africa: the case for increased participation Health Policy and Planning 1996 13 84 92 10.1093/heapol/11.1.84 10155880

[B8] Wegner M Landry E Wilkinson D Tzanis J Men as partners in reproductive health: from issues to action International Family Planning Perspectives 1998 13 38 42 10.2307/2991918

[B9] Coleman WL Garfield CF American Academy of Pediatrics Committee on Psychosocial Aspects of Child and Family Health Fathers and pediatricians: enhancing men's roles in the care and development of their children Pediatrics 2004 13 1406 1411 10.1542/peds.113.5.1406 15121965

[B10] Engle PL The role of men in families: achieving gender equity and supporting children Gender and Development 1997 13 31 40 10.1080/741922351 12292613

[B11] Phares V Lopez E Fields S Kamboukos D Duhig AM Are fathers involved in pediatric psychology research and treatment? J Pediatr Psychol 2005 13 689 693 10.1093/jpepsy/jsi050 15772363

[B12] Robertson LM Douglas F Ludbrook A Reid G van Teijlingen E What works with men? A systematic review of health promoting interventions targeting men BMC Health Services Research 2008 13 141 10.1186/1472-6963-8-141 18598339 PMC2483970

[B13] Barker G Verani F Men's Participation as Fathers in the Latin American and Caribbean Region: A Critical Literature Review with Policy Considerations. Promundo and Save the Children 2008

[B14] Brothers for Life http://www.brothersforlife.org/about_b4l.html

[B15] Sonkhe Gender Justice Network http://www.genderjustice.org.za/

[B16] Mkhize N Morrell R, Richter L African traditions and the social, economic and moral dimensions of fatherhood Baba: Men and Fatherhood in South Africa 2006 Cape Town: HSRC Press 183 198

[B17] Morrell R Morrell R, Richter L Fathers, fatherhood and masculinity in South Africa Baba: Men and Fatherhood in South Africa 2006 Cape Town: HSRC Press 13 25

[B18] Townsend N Tamis-LeMonda CS, Cabrera N Cultural contexts of father involvement Handbook of Father Involvement Multidisciplinary Perspectives 2002 Mahweh, New Jersey: Lawrence Erlbaum Associates, Inc 249 277

[B19] Belsey MA AIDS and the Family: Policy Options for a Crisis in Family Capital 2005 New York: United Nations, Department of Economic and Social Affairs

[B20] Manlove J Logan C Ikramullah E Holcombe E Factors associated with multiple-partner fertility among fathers Journal of Marriage and the Family 2008 13 536 548 10.1111/j.1741-3737.2008.00499.x PMC6738949 31511750

[B21] King V Variation in the consequences of nonresident father involvement for children's well-being Journal of Marriage and the Family 1994 13 963 972 10.2307/353606

[B22] McLanahan S Carlson M Lamb ME Fathers in fragile families The Role of the Father in Child Development 2004 4 New York: John Wiley and Sons 368 396

[B23] Carlson MJ McLanahan S Brooks-Gunn J Coparenting and nonresident fathers' involvement with young children after nonmarital birth Demography 2008 13 461 488 10.1353/dem.0.0007 18613490 PMC2831369

[B24] Casey Foundation Responsible Government: Investing in the Well-Being of Black Fathers, Families and Communities: Julia Carson Responsible Fatherhood and Healthy Families Act of 2009. Position Paper on HR 2979 2009 Seattle: Casey Foundation

[B25] Batty GD Lawlor DA, Mishra GD Family-based life course studies in low- and middle-income countries Family Matters Designing, Analysing, and Understanding Family-based Studies in Life Course Epidemiology 2009 Oxford: Oxford University Press 129 150

[B26] Lawlor DA Mishra GD Lawlor DA, Mishra GD Why family matters: an introduction Family Matters: Designing, Analysing and Understanding Family-based Studies in Life Course Epidemiology 2009 Oxford: Oxford University Press 1 9

[B27] Schoon I Risk and Resilience. Adaptations in Changing Times 2006 Cambridge: Cambridge University Press

[B28] Hosegood V The demographic impact of HIV and AIDS across the family and household life-cycle: implications for efforts to strengthen families in sub-Saharan Africa AIDS Care 2009 13 13 21 10.1080/09540120902923063 22380974 PMC2758218

[B29] Madhavan S DeRose LF Family and Crisis in the Developing World: Implications for Responding to Children Affected by HIV/AIDS Boston: Harvard School of Public Health/JLICA 2008

[B30] Bicego G Rutstein S Johnson K Dimensions of the emerging orphan crisis in sub-Saharan Africa Social Science and Medicine 2003 13 1235 1247 10.1016/S0277-9536(02)00125-9 12600361

[B31] Hosegood V Floyd S Marston M Hill C McGrath N Isingo R Crampin A Zaba B The effects of high HIV prevalence on orphanhood and living arrangements of children in Malawi, Tanzania and South Africa Population Studies 2007 13 327 336 10.1080/00324720701524292 17979006 PMC2216069

[B32] Monasch R Boerma JT Orphanhood and childcare patterns in sub-Saharan Africa: an analysis of national surveys from 40 countries AIDS 2004 13 S55 S65 10.1097/00002030-200406002-00007 15319744

[B33] Case A Paxson C Ableidinger J Orphans in Africa: parental death, poverty and school enrollment Demography 2004 13 483 503 10.1353/dem.2004.0019 15461011

[B34] Rivers J Mason J Silvestre E Gillespie S Mahy M Monasch R Impact of orphanhood on underweight prevalence in sub-Saharan Africa Food Nutr Bull 2008 13 32 42 18510203 10.1177/156482650802900104

[B35] Desmond C Richter L Targeting AIDS orphans and child-headed households? A perspective from national surveys in South Africa AIDS Care 2008 13 1019 1028 10.1080/09540120701842738 18608066 PMC3320103

[B36] Meintjes H Hall K Marera D-H Boulle A Orphans of the AIDS epidemic? The extent, nature and circumstances of child-headed households in South Africa AIDS Care 2010 13 29 39 10.1080/09540120903033029 PMC2840873 20390479

[B37] Locoh T van de Walle E, Sala-Diakanda MD, Ohadike PO Evolution of the family in Africa The State of African Demography 1988 Liege: International Union for the Scientific Study of Population 47 65

[B38] Madhavan S Roy K Securing fatherhood through kinwork: a comparison of low income fathers in South Africa and the U.S. Personalcommunication 10.1177/0192513X11426699 PMC3837197 24273365

[B39] Montgomery C Hosegood V Busza J Timaeus IM Men's involvement in the South African family: engendering change in the AIDS era Social Science and Medicine 2005 13 2411 2419 16300871 10.1016/j.socscimed.2005.10.026

[B40] O'Laughlin B Missing men? The debate over rural poverty and women-headed households in southern Africa The Journal of Peasant Studies 1998 13 1 48

[B41] Townsend N Madhavan S Garey AI Father presence in rural South Africa: historical changes and life-course patterns International Journal of Sociology of the Family 2006 13 173 190

[B42] Townsend NW Men, migration, and households in Botswana: an exploration of connections over time and space Journal of Southern African Studies 1997 13 405 420 10.1080/03057079708708547

[B43] Fitzgerald M Collumbien M Hosegood V "No one can ask me 'Why do you take that stuff?'": men's experiences of antiretroviral treatment in South Africa AIDS Care 2010 13 3 355 360 10.1080/09540120903111536 20390516

[B44] Ramphele M A Bed Called Home: Life in the Migrant Labour Hostels of Cape Town 1993 Cape Town & Johannesburg: David Phillip

[B45] Spiegel A Eades J Dispersing dependents: a response to the exigencies of labour migration in rural Transkei Migrants, Workers and the Social Order 1987 London: Tavistock 113 129

[B46] Budlender D Chobokoane N Simelane S Marriage patterns in South Africa: methodological and substantive issues Southern African Journal of Demography 2005 13 1 25

[B47] Hosegood V McGrath NM Moultrie TA Dispensing with marriage: marital and partnership trends in rural KwaZulu-Natal, South Africa 2000-2006 Demographic Research 2009 13 279 312 10.4054/DemRes.2009.20.13 PMC4340557 25729322

[B48] Preston-Whyte E Women who are not married: fertility, 'illegitimacy', and the nature of households and domestic groups among single African women in Durban South African Journal of Sociology 1993 13 63 71

[B49] Russell M Understanding black households: the problem Social Dynamics 2003 13 5 47

[B50] Measure DHS http://www.measuredhs.com/

[B51] Indepth Network Chapter 1. Core concepts of DSS Population and Health in Developing Countries. Population, Health, and Survival in INDEPTH Sites 2002 13 Ottawa: International Development Research Centre

[B52] Lam D Ardington C Branson N Case A Leibbrandt M Menendez A Seekings J Sparks M The Cape Area Panel Study: A Very Short Introduction to the Integrated Waves 1-2-3-4 Data 2008 Cape Town: The University of Cape Town

[B53] May J Carter MR Haddad L Maluccio JA KwaZulu-Natal Income Dynamics Study (KIDS) 1993-1998: A Longitudinal Household Data Set for South African Policy Analysis CSDS Working Paper No 21 1999

[B54] Richter LM Norris SA De Wet T Transition from birth to ten to birth to twenty: the South African cohort reaches 13 years of age Paediatric and Perinatal Epidemiology 2004 13 290 301 10.1111/j.1365-3016.2004.00572.x 15255883 PMC1885513

[B55] Posel D Devey R Morrell R, Richter L The demographics of fatherhood in South Africa: an analysis of survey data, 1993-2002 Baba: Men and Fatherhood in South Africa 2006 Cape Town: HSRC Press 38 52

[B56] Timæus IM Estimation of mortality from orphanhood in adulthood Demography 1991 13 213 227 10.2307/2061276 2070895

[B57] Madhavan S Fosterage patterns in the age of AIDS: continuity and change Social Science and Medicine 2004 13 1443 1454 10.1016/S0277-9536(03)00341-1 14759688

[B58] Lamb ME Pleck JH Charnov EL Levine JA Lancaster JB, Altman J, Rossi A, Sherrod LR A biosocial perspective on paternal behaviour and involvement Parenting across the Lifespan: Biosocial Dimensions 1987 13 New York: Academic 11 42

[B59] Madhavan S Townsend N The social context of children's nutritional status in rural South Africa Scandinavian Journal of Public Health 2007 13 107 117 10.1080/14034950701355700 17676511 PMC2830101

[B60] Madhavan S Townsend N Garey AI 'Absent breadwinners': Father-child connections and paternal support in rural South Africa Journal of Southern African Studies 2008 13 647 663 10.1080/03057070802259902 20396616 PMC2854816

[B61] The National Income Dynamics Study http://www.nids.uct.ac.za/home/

[B62] Eggebeen DJ Tamis-LeMonda CS, Cabrera N Sociological perspectives on fatherhood: what do we know about fatherhood from social surveys Handbook of Father Involvement: Multidisciplinary Perspectives 2002 Mahweh, New Jersey: Lawrence Erlbaum Associates, Inc 189 209

[B63] Mikelson KS He said, she said: comparing mother and father reports of father involvement Journal of Marriage and Family 2008 13 613 624 10.1111/j.1741-3737.2008.00509.x

[B64] Tomkins S Lawlor DA, Mishra GD Using available family members as proxies to provide information on other family members who are difficult to reach Family Matters Designing, Analysing, and Understanding Family-based Studies in Life Course Epidemiology 2009 Oxford: Oxford University Press 149 179

[B65] Hosegood V Benzler J Solarsh G Population mobility and household dynamics in rural South Africa: implications for demographic and health research Southern African Journal of Demography 2005 13 43 67

[B66] Spiegel AD The fluidity of household composition in Matatiele, Transkei: a methodological problem African Studies 1986 13 17 35 12340942 10.1080/00020188608707648

[B67] Roggman LA Fitzgerald HE Bradley RH Raikes H Tamis-LeMonda CS, Cabrera N Methodological, measurement, and design issues in studying fathers: an interdisciplinary perspective Handbook of Father Involvement: Multidisciplinary Perspectives 2002 Mahweh, New Jersey: Lawrence Erlbaum Associates, Inc

[B68] Rotheram-Borus MJ Flannery E Rice E Lester P Families living with HIV AIDS Care 2005 13 978 987 10.1080/09540120500101690 16176894

[B69] Cabrera N Brooks-Gunn J Moore K West J Boller K Tamis-LeMonda CS Tamis-LeMonda CS, Cabrera N National efforts to learn about fathers Handbook of Father Involvement Multidisciplinary Perspectives 2002 New Jersey: Lawrence Erlbaum Associates, Inc

[B70] Jewkes R Morrell R Gender and sexuality: emerging perspectives from the heterosexual epidemic in South Africa and implications for HIV risk and prevention Journal of the International AIDS Society 2010 13 1 6 20181124 10.1186/1758-2652-13-6 PMC2828994

[B71] King E De Silva M Stein A Patel V Interventions for improving the psychosocial well-being of children affected by HIV and AIDS Cochrane Database of Systematic Reviews 2009 10.1002/14651858.CD006733.pub2 PMC7387107 19370650

